# A Case Report on Oral Subcutaneous Dirofilariasis

**DOI:** 10.1155/2015/648278

**Published:** 2015-12-17

**Authors:** R. D. Jayasinghe, S. R. Gunawardane, M. A. M. Sitheeque, S. Wickramasinghe

**Affiliations:** ^1^Department of Oral Medicine and Periodontology, Faculty of Dental Sciences, University of Peradeniya, 20400 Peradeniya, Sri Lanka; ^2^Department of Oral and Maxillofacial Surgery, Faculty of Dental Sciences, University of Peradeniya, 20400 Peradeniya, Sri Lanka; ^3^Department of Parasitology, Faculty of Medicine, University of Peradeniya, 20400 Peradeniya, Sri Lanka

## Abstract

Dirofilariasis is an uncommon zoonotic parasitic infection affecting human. The natural hosts for this nematode are animals such as dogs, cats, foxes, jackals, and raccoons. This disease is endemic in South Eastern United States, Australia, Europe, and Central and Southern Asia.* Dirofilaria immitis* and* D. repens* are the common mosquito borne filarial nematodes that cause infection. Several species of mosquitos including* Mansonia uniformis*,* M. annulifera*, and* Aedes aegypti* are the potential vectors for this disease in Sri Lanka. Two rare cases of dirofilariasis presenting as facial and intraoral lumps are presented.

## 1. Introduction

Dirofilariasis is an emerging zoonotic parasitic infection caused by a habitual parasite of canines which rarely can cause accidental infections in human beings. In man, dirofilarial infections usually present as pulmonary, peritoneal, ocular, or subcutaneous lesions [1]. Among the 40 species recognized,* Dirofilaria repens, Dirofilaria ursi, Dirofilaria tenuis,* and* Dirofilaria striata* are found in the subcutaneous tissues while* Dirofilaria immitis* and* Dirofilaria spectrum* are found in the heart and blood vessels of man [2]. It is a vector borne disease and transmission to man occurs through the bite of potential mosquito vectors. Exposed part of the body including the head and neck region and the lower extremities form the common subcutaneous site of involvement with the majority of the cases occurring in the ocular and periocular region [3]. We present a rare case of oral dirofilariasis and discuss the various differential diagnosis, clinical, radiologic, and histopathological features and the management.

## 2. Case Report

The following cases presented to the Oral Medicine Clinic of the University Dental Hospital, Peradeniya, Sri Lanka, during a span of four years from July 2011.

### 2.1. Case 1

A 21-year-old male patient presented with a painless nodular swelling on the left cheek of several months duration in July 2011. The lesion measured approximately 1.5 cm in diameter. There was no significant mobility of the nodule under the skin/oral mucosa although it was palpable intraorally as well. Neither the skin nor the mucosa overlying the nodule showed any erythematous appearance. The patient's medical history was nonremarkable and the patient was unable to recall any injury to the face or any insect bite. No larger swelling of the face prior to the development of the nodule was reported. No regional lymph node enlargement was detected.

A differential diagnosis of adenoma arising from minor salivary glands, a fibrosed/calcified lymph node, an inspissated submucosal abscess, and an infected inspissated sebaceous cyst was made. Routine haematological examination revealed no abnormality.

**Figure 1:**
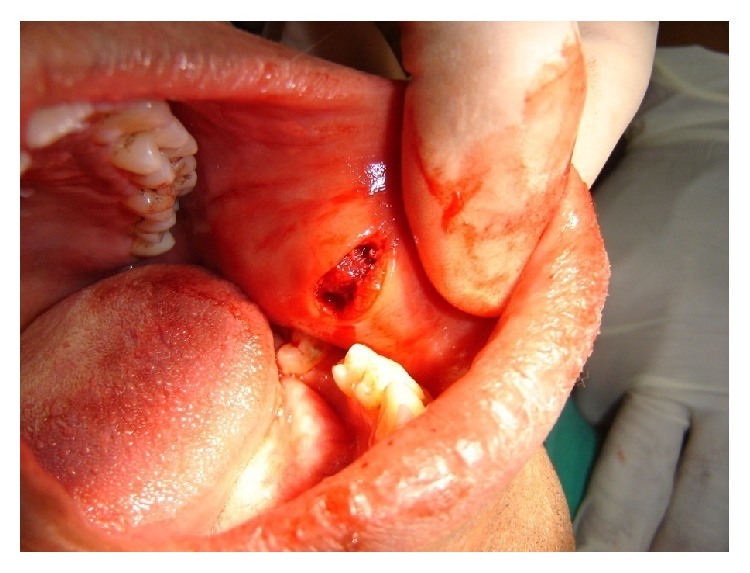
Surgical site after removing the parasite.

**Figure 2:**
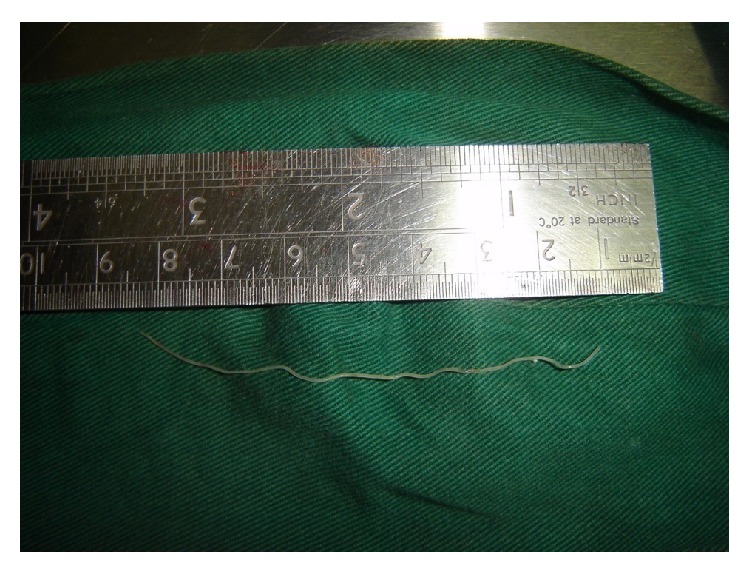
Specimen of* Dirofilaria repens*.

A decision was taken to enucleate the swelling from an intraoral aspect. During the surgery the surface of the nodule was accidentally punctured and a pus-like fluid was found to be oozing from the lesion. It was then decided to open the nodule to drain the remainder of the fluid when a thin ribbon-like object was observed to emerge from the nodule. The remainder of the object was gently evacuated (Figure 1). On becoming apparent the object was a worm it was measured and found to be 7 cm long (Figure 2). It was found to be wriggling for few seconds before it became lifeless. It was immediately sent to the Parasitology Department of the adjoining medical school where it was confirmed to be a male specimen of* Dirofilaria repens*.

The site of surgery healed uneventfully and, on follow-up, no residual lesion was seen.

### 2.2. Case 2

**Figure 3:**
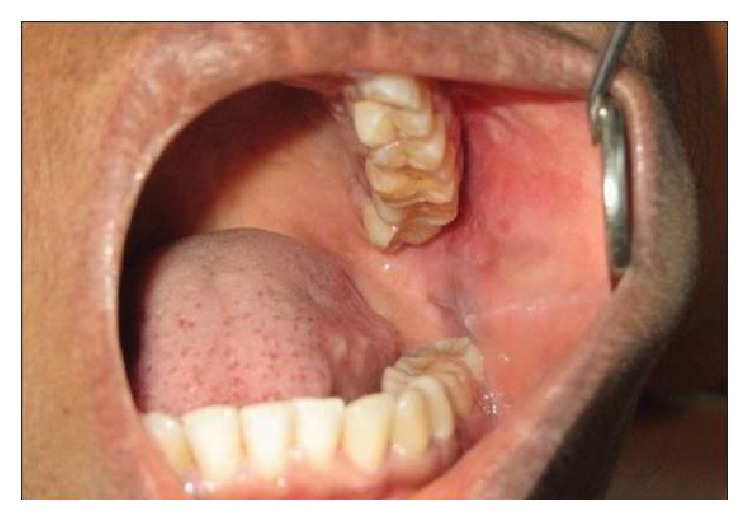
Intraoral appearance.

A 57-year-old female patient with no significant medical history presented to the Dental Hospital Peradeniya, with a mildly tender nodule of eight-month duration on the left side of the cheek. The patient gave a history of a swelling of the left cheek initially with the skin appearing erythematous, which subsequently subsided with the intake of antibiotics only to be followed by the appearance of the nodule. Extra oral examination revealed a firm, nontender nodular swelling on the left cheek, approximately 2 cm × 2 cm in size, palpable under the skin. The margins of the lesion were not well demarcated and the overlying skin appeared normal. No lymphadenopathy was observed. Intraoral examination showed a well circumscribed, partially moveable, firm swelling on the left side buccal mucosa (Figure 3).

**Figure 4:**
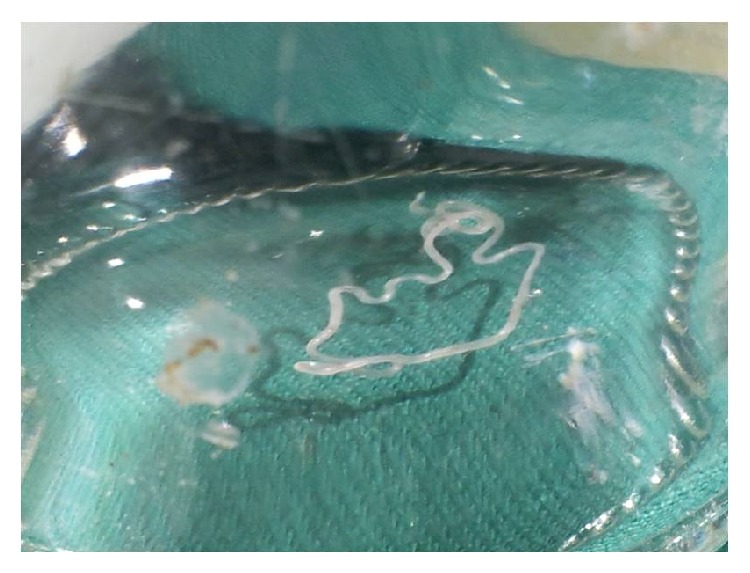
Specimen of* Dirofilaria repens*.

A differential diagnosis as in Case 1 was made. A fine needle aspiration cytology revealed only a chronic inflammatory process. Routine blood examination and biomedical parameters were within normal limits. However in the light of previous experience an ultrasonographic examination of the nodule was sought. The ultrasonograph revealed the presence of a live parasite within the nodule. Subsequently the nodule was excised from an intraoral aspect and the parasite was identified as a female specimen of* Dirofilaria repens* (Figure 4). 

### 2.3. Identification of Worms by SEM

**Figure 5:**
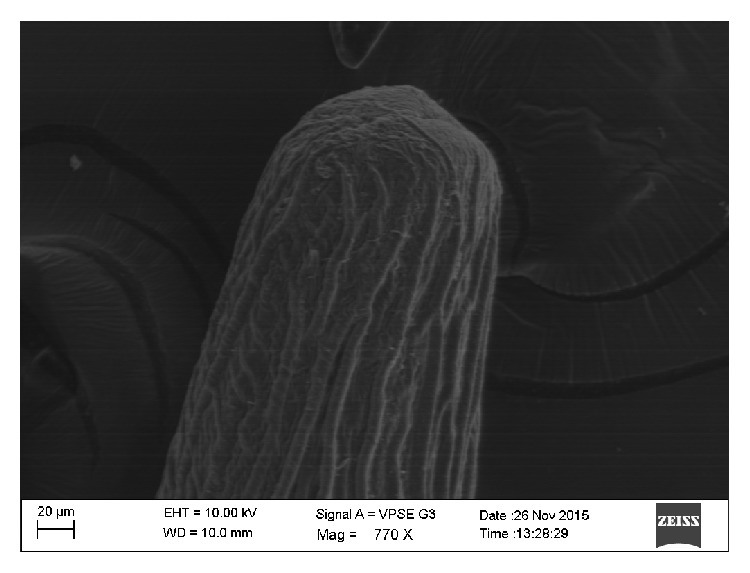
Anterior end of the worm showing thick longitudinal ridges and oral cavity.

**Figure 6 fig6:**
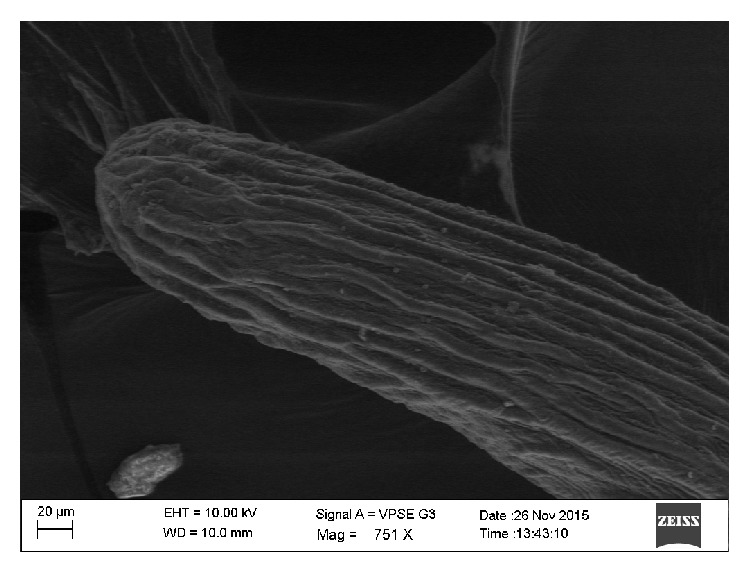
Posterior end of the worm showing thick longitudinal ridges.

**Figure 7 fig7:**
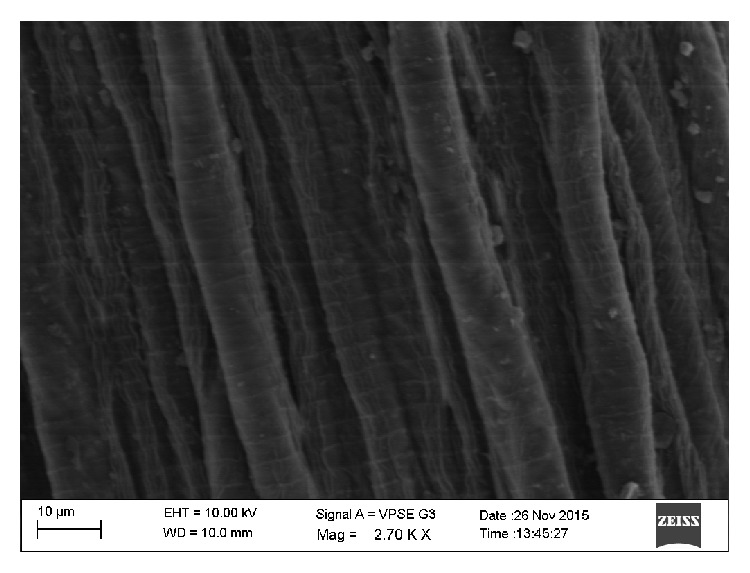
Cuticle of the worm (middle part) showing transverse striations and longitudinal ridges.

**Figure 8:**
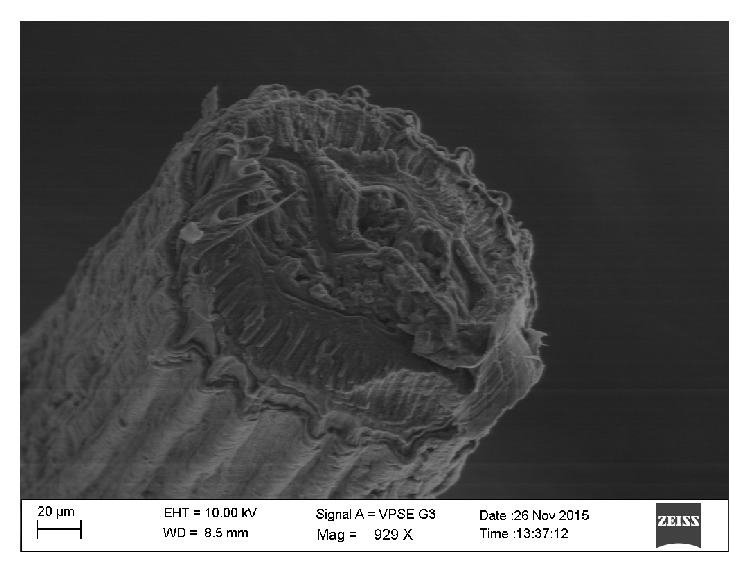
A cross section of the worm showing cuticular ridges and thick musculature.

Two worms were preserved in 70% ethanol (v/v) after isolation from the nodules in the oral cavity. First, samples were scrutinized using a light microscope. Worms were identified using length, width, and appearance of the cuticle. Sex of the worm was determined, based on the presence or absence of vulval opening. Then, samples were further processed for scanning electron microscope (SEM) examination. Briefly, samples were air dried to evaporate ethanol. Next, samples were sectioned into parts (anterior end, middle part, and posterior end) separately. Then, sections were fixed separately on the stub using double side conducting carbon tape. After that samples were loaded into the sputter coater (SC7620, Quorum Technologies, UK) and samples were coated using gold palladium for 60 seconds. Finally, quoted samples were loaded into the scanning electron microscope (EVO/LS15, Carl Zeiss, Germany) and photos were taken at different magnification (Figures 5 [Fig fig6] [Fig fig7]–8).

By histopathological evaluation of the excisional biopsy one of the specimens revealed peripheral layer of granulation tissue with an intense inflammatory cell infiltrate. Center of the lesion showed a nematode parasite exhibiting a thick cuticle with fine external longitudinal ridges and a prominent circumferential muscle layer showing transverse striations.

The site of the nodule healed without any complications or residual abnormality.

## 3. Discussion

Dirofilariasis is a parasite infection of animals and rarely human with nematodes of the genus* Dirofilaria*. Dogs, monkeys, and cats are the primary host and mosquitoes such as* Mansonia uniformis, Mansonia annulifera,* and* Ades aegypti * are considered potential vectors in Sri Lanka [4]. In humans, two types of diseases, namely, pulmonary dirofilariasis, primarily caused by* Dirofilaria immitis,* and subcutaneous dirofilariasis, primarily caused by* Dirofilaria tenuis* and* Dirofilaria repens,* can be seen. In Sri Lanka, the only species that is responsible for human dirofilariasis is* Dirofilaria repens* [1, 4].

The parasite was first recognized by Railliet and Henry in 1911 from a dog and Skrjabin (1917) had described a human case under the name of Loa extraocularis. Skrjabin et al. (1930) attributed a second human case to* Dirofilaria repens* and Skrjabin and Schikhobalova (1948) recognized Loa extraocularis as a synonym of* Dirofilaria repens* [5, 6].

Prevalence of this disease is increasing and emerging as a significant health problem in different areas of the world. The disease is said to be commonly encountered in the 4th to 5th decade of life while showing significant female predilection. Our first case was found in a young male. Significant geographical variation was observed as an endemic in India and Sri Lanka other than Africa, Asia, Australia, Europe, and America [7]. A literature search showed that Tilakaratne and Pitakotuwage (2003) had published a series of seven cases of* D. repens* infection in Sri Lankan patients [8].

The parasite* Dirofilaria repens* is composed of thick external cuticula with longitudinal ridges (microruffling of the surface), leading to a cogwheel-like appearance in a transverse section. Large lateral chords with tall, slender coelomian muscle layer and a single gut tube is also present in this group of parasites. In humans, nodules can occur in any part of the body especially those that are likely sites for mosquito bite. A study of the aetiopathogenesis shows that the adult nematode lives in subcutaneous tissues of their natural hosts and attain full size and deposit microfilaria in blood. Vector arthropods ingest the first stage of larva (L1) while feeding on an infected host and L1 microfilaria develop to infective third stage (L3) and migrate to the proboscis. Transmission occurs when the mosquito vector carrying the infective third stage lava (L3) penetrates a new host, which can be either a human or a natural host. Since the humans are a dead-end host, the nematode in humans does not reach sexual maturity and remains nonfertile. Consequently microfilaria are not released into the peripheral blood in humans and, due to the presence of an adult nematode in the subcutaneous tissue, chronic inflammatory infiltration can occur in the surrounding tissue forming a parasitic granuloma [1, 2, 7, 9].

Clinical features can be seen as single nontender subcutaneous nodule. Mostly the patients are asymptomatic and no particular sensation is attributable to the insect bite. Subsequent formation of subcutaneous or subconjunctival suppurative nodules after 2–12 months can be encountered, or very rarely satellite lymphadenopathies with hyperpyrexia can occur. In the eyes it can cause detached retina, crystalline opacity, glaucoma, uveitis, episcleritis and limited loss of vision. Subcutaneous migration of parasite in tissues of head can cause trigeminal neuralgia [1, 7, 9].

Hematological investigations such as FBC can show peripheral hypereosinophilia as this process has a chronic inflammatory process. Radiological investigations such as computed tomography scan and magnetic resonance imaging may be useful. In this case ultrasonographic imaging showed a worm-like structure that displayed movement inside the cyst and contributed to the diagnosis very significantly. Color Doppler imaging also may be useful in the diagnosis of the disease. Serological investigations such as measuring the reduction in the level of anti-*D. repens *serous antibodies by immunoenzymatic means for 3–6 months from the time of surgery can be used in the follow-up care of patients. Investigations like Giemsa stain and fine needle aspiration cytology followed by excisional biopsy are useful in confirming the diagnosis. Once the excision of the lesion is performed parasitological evaluation can be performed for the worm specimen [1, 9].

As humans are the end hosts, surgical excision of the worm is the appropriate management. Human dirofilariasis is usually regarded as an infection by a single worm. Oral therapy with diethylcarbamazine (DEC) has also been recommended to destroy occult worms [10]. The diagnostic dilemma with subcutaneous/submucosal nodular swellings of the oral cavity is evident from previous reports [11]. Tilakaratne and Pitakotuwage in their series of seven cases gave a variety of differential diagnosis for submucosal nodules without inflammatory signs. The various differential diagnoses given were fibroepithelial polyp, adenoma, salivary gland hyperplasia, lipoma, calcified lymph node, organized mucocele, and tuberculosis [8].

## 4. Conclusion

Even though subcutaneous dirofilariasis is rare, it can be included in the differential diagnosis, when patient presents with a firm submucosal nodule without overlying inflammatory signs which does not completely respond to routine therapy, especially in patients from endemic areas like Sri Lanka.
